# Comment on: The Relationship Between Frailty and Emotional Health in Older Patients With Advanced Cancer

**DOI:** 10.1093/oncolo/oyac151

**Published:** 2022-08-10

**Authors:** Zi-Xiang Chen, Tian Yang

**Affiliations:** Department of Hepatobiliary, Pancreatic and Minimal Invasive Surgery, Zhejiang Provincial People’s Hospital, People’s Hospital of Hangzhou Medical College, Zhejiang, People’s Republic of China; Department of General Surgery, First Affiliated Hospital of Anhui University, Anhui Medical University, Anhui, People’s Republic of China; Department of Hepatobiliary, Pancreatic and Minimal Invasive Surgery, Zhejiang Provincial People’s Hospital, People’s Hospital of Hangzhou Medical College, Zhejiang, People’s Republic of China; Department of Hepatobiliary Surgery, Eastern Hepatobiliary Surgery Hospital, Navy Medical University (Second Military Medical University), Shanghai, People’s Republic of China

We read with great interest the recent article published in *The Oncologist* by Gilmore et al and congratulate the authors on their pioneering work describing the relationship between frailty and emotional health in older patients with advanced cancer.^1^ In this observational study, participants were then stratified based on the Deficit Accumulation Index (DAI) into robust (0 to <0.2), prefrail (0.2 to <0.35), and frail (≥0.35) categories. Multivariate logistic regression models examined the association of frailty with emotional health outcomes. The results revealed that there were age and gender differences among 3 groups (*P* < .05). The mean scores of depression (1.68 vs 2.6 vs 4.86; *P* < .001), anxiety (1.91 vs 2.14 vs 4.6; *P* < .001), and distress (1.9 vs 2.68 vs 3.98; *P* < .001) increased in accordance with the increase of frailty scores (robust vs prefrail vs frail). Furthermore, compared with robust patients, both prefrail and frail patients had an increased risk of screening positive for emotional disorders. Although inspiring and thought-provoking, we would like to raise the following comments.

As showed in Table II of the study by Gilmore et al,^1^ significant differences existed in some variables of patients’ baseline demographic and clinical characteristics, such as age (*P* = .02), gender (*P* = .005), and emotional diseases (*P* < .001) including depression, anxiety, and distress. In addition, cancer type was classified into gastrointestinal, lung, and other, and they were comparable across patients with different frailty statuses (*P* = .47). It is known that people with the same stage of advanced cancer of different prognosis tend to experience different frail stature and different emotional health, such as with pancreatic cancer and breast cancer.^2,3^ In other words, a patient who has an advanced cancer with short-term survival is more likely to develop emotional problem. Herein, we suggest the addition of a variable such as malignant degree (low and high) or prognosis (good and poor) to elucidate the impact of a specific cancer type on the study endpoint.

Another possible flaw of this observational cohort study was the lack of patient’s flow chart,^4^ which prevents readers from getting some important information, including whether all analytic patients were consecutive, how many patients were excluded and the reasons for being excluded, what were the mainstay treatments for advanced cancer, and how many patients were lost to follow up.

In conclusion, the Gilmore et al study is very significant to guide clinical practice for older patients with advanced cancer. We think clarification of the above-mentioned issues would reinforce the validity and credibility of the conclusions of this study.^1^
